# Selection of Promising Candidates for Herbal Veterinary Medicinal Products: A Strategic Approach

**DOI:** 10.1055/a-2891-9080

**Published:** 2026-06-30

**Authors:** Jelena Arsenijević, Céline Renouard, Kristina Bacova, Cigdem Bilgi, Carole Cousin, Antonio Di Loria, Milica Drobac, Frans v. d. Dungen, Georgios Efthimiou, Ivona Foitova, Attila Hunyadi, Anne-Marie Jacques, Olga Kosakowsk, Radoslava Kristofova, Lise Laborieux, Mirjana Marčetić, Monika Pogány Simonova, Zeynep Semen, Sylwia Styczyńska, Theresa Schlittenlacher, Liliana Vargas-Murga, Michael Walkenhorst, Begum Yurdakok-Dikmen, Marta Mendel

**Affiliations:** 1 University of Belgrade – Faculty of Pharmacy, Department of Pharmacognosy, Belgrade, Serbia; 2 French Agency for Food, Environmental and Occupational Health and Safety, Javené, Fougéres, France; 3 87171 Centre of Biosciences, Slovak Academy of Sciences, Institute of Animal Physiology, Department of Digestive Tract Physiology, Kosice, Slovakia; 4 Zafer Pharmacy, Urla, İzmir, Turkiye; 5 Department of Veterinary Medicine and Animals Productions, University of Napoli Federico II, Napoli, Italy; 6 ENDC B. V., Harderwijk, Netherlands; 7 Centre for Biomedicine, Hull York Medical School, Hull, United Kingdom; 8 Masaryk University, Faculty of Science, Department of Botany and Zoology, Brno, Czech Republic; 9 Institute of Pharmacognosy, University of Szeged, Szeged, Hungary; HUN-REN-SZTE Biologically Active Natural Products Research Group, Szeged, Hungary; 10 Warsaw University of Life Sciences, Institute of Horticultural Sciences, Department of Vegetable and Medicinal Plants, Warsaw, Poland; 11 Dokuz Eylül University, Faculty of Veterinary Medicine, Department of Biochemistry, İzmir, Turkiye; 12 Research Institute of Organic Agriculture (FiBL), Frick, Switzerland; 13 Department of Chemical and Agricultural Engineering and Agrifood Technology, University of Girona (UdG), Girona, Catalonia, Spain; 14 Ankara University, Faculty of Veterinary Medicine, Department of Pharmacology and Toxicology, Ankara, Turkiye; 15 Warsaw University of Life Sciences, Institute of Veterinary Medicine, Department of Preclinical Sciences, Warsaw, Poland

**Keywords:** medicinal plants, herbal veterinary medicinal products, legal framework

## Abstract

The growing urgency to reduce antimicrobial and antiparasitic use in livestock has renewed scientific interest in medicinal plants as sustainable components of veterinary healthcare. However, the absence of a dedicated regulatory framework for herbal veterinary medicinal products (HVMPs) in the European Union continues to hinder their wider application.

The need for a science-based, multi-criteria framework to identify medicinal plant species as sources of active substances of herbal origin, with the highest potential for future inclusion in a dedicated EU registration pathway for HVMPs, resulted in the establishment of a multidisciplinary expert panel. The panel evaluated over 100 medicinal plant species with documented veterinary application, using a structured scoring system integrating regulatory, pharmacopoeial, toxicological, and sustainability sources. These included regulations and documents related to veterinary, pharmaceutical, food, and feed data, as well as considerations for conservation. Weighted scores reflecting all these aspects were applied to rank the species.

The assessment resulted in the creation of List A, which contains the most promising candidates for future HVMPs, and List B, which compiles plant species whose simplified registration is currently not considered a priority or is not advisable based on the available data. Eighty-three species received positive overall scores. These medicinal plants demonstrate strong regulatory recognition, pharmacopoeial inclusion, and well-documented safety profiles, highlighting their suitability for evidence-based veterinary use.

The proposed framework offers a clear, reproducible method for prioritizing botanical species for regulatory consideration as HVMPs. It supports EU initiatives to enhance sustainable animal health, antimicrobial stewardship, and innovation in veterinary phytotherapy.

**Abbreviations**
AMR: antimicrobial resistance
ANSM: Agence Nationale de Sécurité du Médicament et des Produits de Santé
CAP: Common Agricultural Policy
CVMP: Committee for Veterinary Medicinal Products
EFSA: European Food Safety Authority
EMA: European Medicines Agency
ESCOP: European Scientific Cooperative on Phytotherapy
FAO: The Food and Agriculture Organization
FASFC: Federal Agency for the Safety of the Food Chain
GMOs: genetically modified organisms
HMP: Herbal Medicinal Product
HMPC: The Committee on Herbal Medicinal Products
HVMP: Herbal Veterinary Medicinal Products
IUCN: International Union for Conservation of Nature
JECFA: Joint FAO/WHO Expert Committee on Food Additives
MRL: maximum residue limit
WHO: World Health Organization

## Introduction

In light of evolving European policy frameworks and shifting clinical paradigms, there is a growing imperative to reduce reliance on antimicrobials and antiparasitics in veterinary medicine while safeguarding animal health and welfare 1 , 2 . While antibiotics and anthelminthics have long been central to the treatment of infectious and invasive diseases in animals–thereby improving livestock health, welfare, and productivity–their extensive and, at times, indiscriminate use has accelerated the emergence of resistant microbial strains and reduced their effectiveness 3 – 6 .

This imperative is reinforced by several key policies and scientific drivers. First, the European Green Deal and its Farm to Fork Strategy have explicitly prioritized mitigating antimicrobial resistance (AMR), setting an ambitious target of a 50% reduction in overall antimicrobial sales for livestock and aquaculture within the EU by 2030. To operationalize this objective, EU member states have embedded targeted AMR reduction measures within their Common Agricultural Policy (CAP) Strategic Plans, integrating health, welfare, good management practices, and sustainability goals into national agricultural frameworks 7 . Moreover, the European Commissionʼs aim to transition 25% of agricultural land to organic farming underscores a broader agroecological transformation, in which antimicrobial, antiparasitic, anti-inflammatory, and other conventional drugs ^1^ are inherently restricted, and phytotherapy is gaining greater attention 8 . This transition is further supported by complementary initiatives, including the Zero Pollution Action Plan 9 , the Strategic Approach to Pharmaceuticals in the Environment 10 , the new WHO Traditional Medicine Strategy 2025 – 2034 11 and the consolidation of the One Health framework that advocates for an integrated, cross-sectoral approach to managing health at the human–animal–environment interface and the cooperation of experts across disciplines 12 . Simultaneously, scientific and clinical interest in evidence-based phytotherapy is increasing. Data generated in recent studies support the growing use of these modalities in veterinary practice, particularly in the context of chronic conditions, antimicrobial stewardship, and integrative animal care 13 – 18 . Concurrently, there is an observable shift in behavior among animal owners–especially in the companion animal sector–toward the use of herbal and natural remedies. This trend is often driven by perceptions of improved safety, accessibility, and cost-effectiveness, as well as a preference for self-managed care 19 . At the same time, conventional veterinary drugs, including antiparasitic agents, pose additional ecological risks: because only a small fraction of the administered dose is metabolized within the treated animal, it is estimated that 80 – 98% of the active compound is excreted unmetabolized 20 , 21 . These residues subsequently enter the environment either directly via animal excreta or indirectly through manure application, where they can accumulate in soil ecosystems and exert toxic effects, particularly on coprophagous and soil-dwelling invertebrates, in some cases with fatal consequences for sensitive species 22 – 25 . Collectively, these developments underscore the urgent need for integrative strategies that reduce dependency on antibiotics and antiparasitics at their source. This can be achieved through improved disease prevention, enhanced husbandry practices, and the evidence-based deployment of complementary and alternative therapeutic modalities with the ultimate aim of promoting resilient health systems that align with both veterinary public health priorities and the EUʼs broader sustainability agenda.

Among the alternatives, veterinary phytotherapy has garnered renewed scientific interest. Medicinal plants, for example, offer antimicrobial, anti-inflammatory, immunomodulatory, and digestive-supportive properties that can help reduce the use of conventional drugs in animal husbandry 14 , 26 , 27 . For a long time, and recently, they have been used by animal keepers and veterinarians to treat and prevent animal diseases 27 . Recent studies have demonstrated the efficacy of specific plant constituents, such as essential oils, tannins, saponins, and polyphenols, in inhibiting pathogenic bacteria, reducing endoparasites, and modulating the host immune response 28 – 31 , as well as growth promoters and gut health modulators, with promising outcomes particularly in monogastric species 32 – 34 . Various products derived from medicinal plants can be authorized for use in the European market as feed, feed additives, biocides, animal healthcare products, and, recently, of particular regulatory interest, herbal veterinary medicinal products (HVMP).

### Current legal situation of HVMPs in Europe

Within the European Union, the availability of HVMPs for veterinary practices is insufficiently regulated by a comprehensive, evolving regulatory and policy framework. Regulation (EU) 2019/6 on veterinary medicinal products–fully applicable since January 2022–provides a harmonized legal foundation for the authorization, marketing, and pharmacovigilance of veterinary medicinal products, including those of herbal origin 35 . This legislation is further complemented by Regulation (EU) 2019/4 on medicated feed 36 and strategically underpinned by the EU One Health Action Plan against Antimicrobial Resistance (AMR), which promotes prudent use of antimicrobials across human and veterinary medicine 37 . Under Article 141 of Regulation (EU) 2019/6, the European Medicines Agency (EMA) Committee for Veterinary Medicinal Products (CVMP) plays a vital role in the authorization, pharmacovigilance, and risk management of veterinary medicines in the European Union, including the centralized procedure and harmonization process. Furthermore, EMA, through its Committee on Herbal Medicinal Products (HMPC), plays a key role in advancing the scientific assessment of herbal substances and herbal preparations. This includes the development of European Union monographs on herbal substances/preparations for human use, but not specific regulatory guidance to ensure the quality, safety, and efficacy of HVMPs in veterinary settings. The establishment of EU herbal monographs and list entries is coordinated by the EMAʼs HMPC, which conducts assessments following a defined procedure. At various stages, stakeholders may contribute by proposing substances, providing scientific data, or commenting during public consultations. The HMPC also prioritizes which herbal substances or preparations should be assessed, with the current proposals published in dedicated inventories. Notably, the inventory of herbal substances for assessment was last updated in July 2021 38 . Despite their therapeutic potential and growing interest, HVMPs face significant regulatory and scientific challenges. Chief among these are issues related to the standardization of herbal substances and preparations, ensuring batch-to-batch consistency, and generating robust clinical data that meet the evidentiary standards required for market authorization and registration. Nevertheless, increasing political and scientific momentum, driven by the AMR agenda and the EUʼs broader sustainability objectives articulated in the Farm to Fork Strategy and the European Green Deal 39 , has created a more favorable environment for the development and uptake of HVMPs as part of a holistic and sustainable model of animal health management. However, existing EU legislation is not yet fully equipped to support the streamlined regulation of HVMPs. As acknowledged in the preamble to Regulation (EU) 2019/6 (Preamble 12), there is a pressing regulatory need to establish simplified procedures tailored specifically to (traditional) herbal veterinary products, but a knowledge gap remains. Addressing this gap is a central objective of the MedPlants4Vet initiative (CA22109, COST Action entitled Medicinal plants for animal health care: Translating tradition into modern veterinary medicine) 40 , which aims to facilitate the creation of a dedicated regulatory pathway for HVMPs. To support this objective, a multidisciplinary panel of experts in phytochemistry, pharmacognosy, quality assurance, veterinary pharmacology and toxicology, safety assessment, and sustainability conducted a structured evaluation of more than 100 relevant medicinal plant species as botanical sources of corresponding active substances of herbal origin (herbal substances/preparations). This perspective was adopted because many of the regulatory and toxicological data sources used in this assessment are indexed by plant species, thereby enabling a more comprehensive evaluation of both their therapeutic potential and the associated risks of use. This exemplary assessment aimed to test and validate a methodological framework for categorizing plants into those with high potential for use as herbal starting material in the production of herbal substances/preparations intended as active substances in future HVMPs and those with conditional or limited applicability.

The initial identification of plant species was conducted by experts within project CA22109 (as of September 2024). All plant species included in the evaluation were selected based on documented traditional use, as evidenced by ethnoveterinary studies, historical textbooks, or their inclusion in recently or previously marketed HVMPs 27 , 41 – 43 , CA22109 inventory of recently or previously marketed HVMPs]. Notably, the fact that they were marked as HVMPs in various regions of Europe confirms the clinical experience documented in their dossiers, supporting their safety and efficacy. The evaluation, grounded in relevant European and national legislative frameworks and scientific guidelines, resulted in the categorization of the plant species into two lists: (A) a list of the most promising candidates for simplified registration as HVMP, and (B) a list of less promising candidates, based on current regulatory, scientific, and technical considerations. Notably, the proposed assessment methodology is primarily oriented toward consumer safety and is therefore designed to evaluate medicinal plants as potential candidates for the development of HVMPs intended for food-producing animals. This approach operates under the assumption that plants deemed safe for human use are unlikely to pose significant risks to treated animals, particularly herbivores and omnivores. However, it is essential to clarify that this methodology is not directly applicable to companion animals, such as dogs and, especially, cats, where consumer exposure is not a concern, but species-specific safety becomes paramount. In such cases, the toxicological assessment must focus on the animalʼs physiological and metabolic responses to phytochemicals. Particular caution is warranted in felines, as they exhibit distinct metabolic limitations, including deficiencies in hepatic detoxification pathways, which render them especially susceptible–or even intolerant–to various pharmacologically active compounds, including those derived from plants 44 .

### Multi-step procedure of the assessment of the medicinal plantsʼ candidates for herbal veterinary medicinal products

The evaluation and subsequent classification of plant species into Lists A and B were conducted in accordance with the relevant provisions of the following legal frameworks, regulatory guidelines, and authoritative databases ( Table 1 ).

**Table:** **Table 1** Legal documents, monographs, and databases used for the assessment.

Data type	Legal framework/database
Veterinary data	Commission Regulation (EU) No. 37/2010 of 22 December 2009 on pharmacologically active substances and their classification regarding maximum residue limits in foodstuffs of animal origin
Pharmaceutical data	European Pharmacopoeia
	European Medicines Agency herbal monographs/Committee on Herbal Medicinal Products (HMPC)
	WHO monographs
	European Scientific Cooperative on Phytotherapy (ESCOP) Monographs
	List of traditionally used medicinal plants according to the French National Agency for Medicines and Health Products Safety (Agence Nationale de Sécurité du Médicament et des Produits de Santé)
Food and feed data	Commission Regulation (EU) 2017/1017 of 15 June 2017 amending Regulation (EU) No. 68/2013 on the Catalogue of feed materials
	Codex alimentarius
	Food and Feed Information Portal Database
	EFSA Compendium of Botanicals – Botanical Summary Report
	BelFrIt List of plants allowed in food supplements
	ComplʼAlim: French Food Supplements Database
	Appendix List 1 of Belgian Royal Decree of 31 August 2021, i.e., list of plants not allowed for use in foodstuffs
	Annex I of Regulation (EC) No. 396/2005 on products of plant (or animal) origin included in the regular human diet
Conservation status	UICN Red List

### Assessment elements

#### Commission Regulation (EU) No. 37/2010 of 22 December 2009 on pharmacologically active substances and their classification regarding maximum residue limits in foodstuffs of animal origin

Commission Regulation (EU) No. 37/2010 establishes a harmonized, consolidated list of pharmacologically active substances authorized for use in food-producing animals, along with their corresponding MRLs for specific edible tissues and products, including muscle, liver, kidney, fat, milk, eggs, and honey 45 . The primary objective of the regulation is to ensure that residues of veterinary medicinal products in foodstuffs derived from treated animals do not pose a risk to public health. In accordance with the regulation, all pharmacologically active substances present in a veterinary medicinal product intended for use in food-producing animals must be included in Annex Table 1 of Regulation (EU) No. 37/2010. Interestingly, the regulation encompasses not only chemically defined single compounds but also herbal substances and herbal preparations (e.g., essential oils and extracts), as well as herbal substances used in homeopathic preparations. Substances not listed in this table either do not fall within the scope of Regulation (EC) No. 470/2009 of the European Parliament and of the Council of 6 May 2009 laying down Community procedures for the establishment of residue limits of pharmacologically active substances in foodstuffs of animal origin, such as non-active excipients 46 , or must undergo a scientific assessment by the European Medicines Agencyʼs Committee for Veterinary Medicinal Products (CVMP). This evaluation encompasses the safety of the substance and its residues, culminating in the issuance of a scientific opinion on MRLs. In the context of the current assessment, particular attention was given to the information provided in the column of the Annex Table 1 entitled “Other Provisions” (according to Article 14(7) of Regulation (EC) No. 470/2009). These additional provisions often impose restrictions on the conditions of use of the substance in veterinary medicinal products, such as limitations on the route of administration or specifications that the substance may be used solely in homeopathic preparations under defined conditions. However, from a consumer safety perspective, where Annex Table 1 indicates that a substance is “for use in homeopathic veterinary medicinal products prepared according to homeopathic pharmacopoeias at concentrations corresponding to dilutions of the mother tincture only”, such restrictions do not exclude its application within phytotherapeutic treatment paradigms. It is noteworthy that, as of the current version of Regulation (EU) No. 37/2010, only a limited number of substances of herbal origin are included in Annex Table 2 , which lists substances strictly prohibited in food-producing animals pursuant to Article 5 of Regulation (EC) No. 470/2009. Specifically, this prohibition currently applies only to the genus Aristolochia and all preparations derived therefrom due to their documented toxicological risks. As part of the present assessment, a scoring system was applied to evaluate the regulatory status of each substance listed under Regulation (EU) No. 37/2010. Substances included in Annex Table 1 without restrictions were assigned a score of 2, indicating full regulatory approval for use in food-producing animals. Substances listed in Annex Table 1 with the restriction “for topical use only” or “for use of homeopathic mother tincture and dilutions thereof” received a score of 1, reflecting limited applicability. Substances annotated exclusively with “for use in homeopathic preparations in dilutions only”, as well as those not listed at all in the regulation, were assigned a score of 0 ( Table 2 ).

**Table:** **Table 2** Scoring system used to assess the candidates for HVMP.

Assessment criteria	Score
** Presence of the plant or plant preparation in Table 1 of the Commission Regulation (EU) No. 37/2010 of 22 December 2009 on pharmacologically active substances and their classification regarding maximum residue limits in foodstuffs of animal origin, verification of the “Other provisions” column **	
yes	2
yes, for external use	1
yes, in case of use of homeopathic mother tincture and dilutions thereof	1
yes, in case of use of homeopathic preparations in dilutions only	0
no	0
**Presence of European Pharmacopoeia 11th Edition:**	
yes	1
no	0
**Existence of HMPC/EMA monograph:**	
yes	2
no	0
**Existence of the WHO monograph:**	
yes	1
no	0
**Existence of the ESCOP monograph:**	
yes	1
no	0
**Categorization of medicinal plants according to the French National Agency for Medicines and Health Products Safety:**	
list A	2
list B	− 1
not listed	0
**Listed in the Catalogue of Feed Materials (Commission Regulation (EU) 2017/1017)**	
yes	1
no	0
**Presence on one of the Codex Alimentarius lists:**	
yes	1
no	0
**Classification in the Food and Feed Information Portal Database:**	
authorized for use in food (i.e., as a food additive and/or food flavoring and/or novel food)	1
authorized as feed additives	2
for both of the above uses	3
forbidden as a feed additive	− 2
n/a	0
**Notifications in the EFSA Compendium of Botanicals – Botanical Summary Report:**	
contains chemicals of concern	− 1
adverse effects not associated with chemicals of concern reported	− 1
contains chemicals of concern and adverse effects not associated with them reported	− 2
n/a	0
**Presence on the BelFrIt List of plants allowed in food supplements:**	
yes	1
no	0
**Status in the ComplʼAlim French database on food supplement:**	
the plant and the conditions of use are authorized	1
the plant and the condition of use are unauthorized	− 1
n/a	0
**Presence on List 1 of the Belgian Plants Order:**	
yes	− 1
no	0
**Presence in Annex I of Regulation (EC) No. 396/2005:**	
yes	− 1
no	0
**UICN Red List status:**	
not evaluated or data deficient	0
least concern	1
near threatened or vulnerable	− 1
endangered or critically endangered	− 2
extinct in the wild or extinct	− 3

#### European pharmacopoeia

The European Pharmacopoeia serves as the single authoritative reference for the quality control of medicinal products in the signatory states of the Convention on the Elaboration of a European Pharmacopoeia 47 . The standards published in the compendium have both legal and scientific authority, serving as the basis for quality assurance throughout the development, manufacturing, and marketing of medicinal products, including herbal substances. Its primary objective is to safeguard public health by establishing harmonized, scientifically validated standards that ensure the quality, safety, and efficacy of medicinal products and their constituents. These standards are designed to support the safe and effective use of medicines by patients while also facilitating the free movement of medicinal products within the European market and globally. The monographs and general chapters of the European Pharmacopoeia are developed to meet the practical and regulatory needs of the following: (a) regulatory authorities, for the evaluation and oversight of pharmaceutical quality; (b) quality control laboratories, responsible for the testing and verification of medicinal products and raw materials; and (c) pharmaceutical manufacturers, engaged in the formulation and production of medicines and their individual components. In an increasingly globalized pharmaceutical environment, where international trade and regulatory harmonization are of growing importance, the European Pharmacopoeia is not only extensively used across Europe but is also recognized and applied internationally. The scope of the European Pharmacopoeia encompasses the analytical procedures and acceptance criteria applied to finished products, starting materials, and synthesis intermediates. Consequently, all manufacturers of medicinal products or substances intended for pharmaceutical use are required to comply with these standards as a prerequisite for market authorization and distribution within the territories of the conventionʼs signatories. As part of the present evaluation, herbal substances ^2^ included in the 11th edition of the European Pharmacopoeia, which was in force at the time the evaluation was conducted, were assigned a score of 1, whereas those not listed were assigned a score of 0 ( Table 2 ).

#### European medicines agency database/Committee on herbal medicinal products

The European Medicines Agency (EMA) operates a range of databases and information systems that facilitate the regulation, safety surveillance, and transparency of medicinal products within the European Union. Among its scientific committees, the Committee on Herbal Medicinal Products (HMPC), established under Directive 2004/24/EC, plays a pivotal role in the EUʼs regulatory framework for herbal medicinal products intended for human use. The EU herbal monographs, developed by the HMPC, provide comprehensive information required for the regulatory approval of herbal medicinal products 48 . This includes therapeutic indications, dosage recommendations, intended target population, and safety-related data such as adverse reactions and known interactions with other medicinal products. These monographs serve as the scientific foundation for product-specific documentation, including the summary of product characteristics and the package leaflet. Each monograph is published alongside supporting documentation, particularly a public assessment report that summarizes the evaluation of available data on the medicinal use of the respective herbal substance/preparation. These monographs may be cited in marketing authorization applications (in the context of well-established use) or in traditional-use registration dossiers. While member states are expected to consider the final monographs during product evaluation, divergence from HMPC-adopted content must be scientifically justified. Additionally, based on the HMPCʼs scientific assessments, the European Commission Decision 2008/911/EC established a list of herbal substances, preparations, and combinations (“list entries”) suitable for use in certain traditional herbal medicinal products. Unlike EU monographs, they are legally binding on all relevant actors in the member states. In the context of the present evaluation, it was verified whether a given herbal substance was the subject of an HMPC monograph available on the EMA website. If a monograph was identified, a score of 2 points was assigned; in the absence of a monograph, a score of 0 was recorded ( Table 2 ). Additionally, it was noted whether the monograph supports well-established use or traditional use.

#### World Health Organization monographs

The WHO Monographs on Selected Medicinal Plants are authoritative reference documents issued by the World Health Organization that provide scientifically substantiated data on the safety, efficacy, and quality control parameters of selected medicinal plant species 49 – 52 . These monographs function as globally recognized standards intended to support national and regional regulatory authorities, healthcare professionals, researchers, and manufacturers engaged in the evaluation, development, and regulation of herbal medicinal products. Particularly valuable for countries lacking comprehensive national pharmacopoeias or established monograph systems, the WHO monographs contribute to the establishment of evidence-based policies and regulatory frameworks for traditional and herbal medicines. Between 1999 and 2009, the WHO published a series of volumes encompassing detailed profiles of the most widely used medicinal plants worldwide. While these monographs do not constitute pharmacopoeial standards per se, they offer rigorous scientific evaluations designed to guide regulatory decision-making and clinical use. For the purposes of the present evaluation, a binary scoring system was applied ( Table 2 ): a score of 1 was assigned if a WHO monograph was available for the medicinal plant under review and a score of 0 if no such monograph existed.

#### European Scientific Cooperative on Phytotherapy (ESCOP) monographs

The ESCOP Monographs are authoritative scientific documents developed by the European Scientific Cooperative on Phytotherapy (ESCOP) to provide comprehensive, evidence-based evaluations of herbal substances and preparations 53 . These monographs are intended to facilitate the safe, rational, and effective use of herbal medicinal products within European and national regulatory frameworks. Each monograph systematically presents information on the botanical identity of the plant material, the specific plant part used, known active constituents, therapeutic indications, dosing regimen, routes of administration, pharmacological data, available clinical efficacy data, and a detailed safety profile. ESCOP monographs serve as recognized scientific references for regulatory agencies, healthcare professionals, researchers, and pharmaceutical stakeholders engaged in the assessment and development of phytotherapeutic products. In the context of the present evaluation, the presence of a published ESCOP monograph for a given herbal substance was assigned a score of 1, while its absence was scored as 0 ( Table 2 ).

#### List of traditionally used medicinal plants according to the French National Agency for Medicines and Health Products Safety (Agence Nationale de Sécurité du Médicament et des Produits de Santé)

The List of Traditionally Used Medicinal Plants 54 , established by the French National Agency for Medicines and Health Products Safety (Agence Nationale de Sécurité du Médicament et des Produits de Santé, ANSM), constitutes an official regulatory instrument embedded in the French Pharmacopoeia. This list, referred to as List A, includes plant species and their corresponding herbal drugs and preparations that fulfill the criteria for traditional medicinal use, namely a documented history of use for at least 30 years, including at least 15 years within the European Union, for the same therapeutic indication. The primary objective of List A is to facilitate the simplified registration procedure for traditional herbal medicinal products, as defined under Directive 2004/24/EC. Inclusion on List A signifies that a medicinal plant is supported by adequate documentation attesting to its safety and prolonged medicinal use, although clinical efficacy derived from contemporary randomized controlled trials may not be available or required. This regulatory tool thereby supports national- and EU-level harmonization of safety standards for traditional herbal medicines. In parallel, List B–also maintained by ANSM–comprises plant taxa considered to present a potentially unfavorable benefit–risk profile, when used either in unprocessed forms or preparations. Plants included in List B are typically associated with inherent toxicity or pharmacological risks (e.g., *Aconitum sp.* and *Ephedra sp.* ). Inclusion in List B generally precludes simplified registration and necessitates stricter regulatory scrutiny. For the purposes of the current evaluation, a quantitative scoring system was applied based on the presence of substances in these prioritization lists. Specifically, medicinal plants included in List A were assigned a score of 2, reflecting high pharmacopoeial relevance. Medicinal plants listed in List B received a score of − 1, denoting lower regulatory priority. Medicinal plants not appearing in either list were assigned a neutral score of 0, indicating either insufficient information or a lack of current consideration within the pharmacopoeial framework ( Table 2 ).

#### Commission regulation (EU) 2017/1017 – Catalogue of feed materials

The Catalogue of Feed Materials, as outlined in Commission Regulation (EU) 2017/1017, is an EU-wide reference list of raw materials that can be used in animal feed, along with their definitions, compositional requirements, and labeling rules 55 . Although its requirements are not obligatory, feed business operators are legally required to comply with the corresponding definition and conditions when labeling a product with a name listed in the catalogue. This ensures that the same feed material is understood consistently across the EU, improving transparency, safety, and fair trade. The regulation provides names and descriptions of feed materials and processing methods, sets purity and safety standards (including maximum levels for undesirable substances, botanical purity thresholds, and impurity limits), mandatory labeling particulars (e.g., moisture content, processing conditions), as well as special provisions for certain categories. For the purposes of the present evaluation, plant species recognized as a feed material in the catalogue were assigned a score of 1, whereas species absent from these lists received a score of 0 ( Table 2 ).

#### Codex Alimentarius

The Codex Alimentarius represents a joint intergovernmental initiative established by the Food and Agriculture Organization (FAO) of the United Nations and the World Health Organization (WHO), functioning as the principal global reference for the development of harmonized food standards, guidelines, and codes of practice 56 . Its overarching objective is to safeguard consumer health and facilitate equitable practices in international food trade. Governance of the Codex Alimentarius Programme is vested in the Codex Alimentarius Commission, which comprises member states and observer organizations and operates through a network of specialized technical committees and task forces. A pivotal instrument within the Codex framework is the “Classification of Food and Animal Feed Products”, developed to support the establishment of maximum residue limits for pesticides, veterinary medicinal products, and other chemical contaminants. This classification system systematically assigns unique numerical codes and standardized nomenclature to individual commodities or commodity groups, thereby ensuring uniformity and coherence in risk assessment procedures and regulatory oversight among member countries. The classification scheme is hierarchically organized, encompassing primary food commodities–including fruits, vegetables, cereals, meat, and milk–processed food products, and animal feedstuffs. Commodities are categorized based on their origin (plant or animal), anatomical part, or intended use. This structured approach enables expert bodies such as the Joint FAO/WHO Expert Committee on Food Additives (JECFA) to formulate commodity-specific residue definitions, analytical methodologies, and MRL recommendations. While the Codex Alimentarius does not directly regulate herbal medicinal products, numerous herbal preparations are incorporated within the food classification system when designated for nutritional, supplementary, or traditional dietary purposes. Consequently, the Codex framework influences international regulatory landscapes, particularly in contexts where the demarcation between food and medicinal products is ambiguous and subject to differential oversight. For the purposes of the present evaluation, plant species documented in any of the Codex classification lists were assigned a score of 1, whereas species absent from these lists received a score of 0 ( Table 2 ).

#### Food and feed information portal database

The Food and Feed Information Portal is an online platform maintained by the European Food Safety Authority (EFSA) 57 . It serves as a centralized access point to a wide range of scientific data, regulatory tools, and databases relevant to food and feed safety within the European Union. The primary objective of the portal is to enhance transparency, data harmonization, and accessibility, thereby supporting risk assessment processes and informed regulatory decision-making. The portal encompasses resources relevant to multiple domains, including food and feed safety (e.g., contaminants, residues, and additives), nutrition and health claims, animal health and welfare, pesticide residues and maximum residue limits, plant health, and genetically modified organisms (GMOs). By consolidating and standardizing key information, the platform facilitates evidence-based risk assessments and supports harmonized regulatory actions across EU member states. As part of the current evaluation concerning the potential of medicinal plants as candidates for future HVMPs, the presence of a given plant species in the platform was verified, along with its regulatory status. This included data such as the expiration date of relevant authorizations and the categories of animals for which the use is permitted. A scoring system was applied as follows ( Table 2 ): plants authorized for use in food (e.g., as food additives, flavorings, or novel foods): 1 point; plants authorized for use as feed additives: 2 points; plants authorized for both food and feed applications: 3 points; plants explicitly prohibited as feed additives: − 2 points.

#### EFSA Compendium of Botanicals – Botanical summary report

The EFSA Compendium of Botanicals, officially titled the Compendium of Botanicals Reported to Contain Naturally Occurring Substances of Possible Concern for Human Health When Used in Food and Food Supplements, is a scientific resource developed and maintained by the European Food Safety Authority 58 . It is specifically designed to support the hazard identification stage of the risk assessment process for botanicals and botanical preparations intended for use in foodstuffs, including dietary supplements. This compendium consolidates data from a wide array of authoritative sources, such as national monographs, international regulatory reports, peer-reviewed scientific literature, and safety assessments. It catalogues plant species reported to contain naturally occurring substances that may pose a potential risk to human health under specific conditions of exposure (such as certain alkaloids, components of essential oils, coumarins, etc.). Identified concerns pertain to a range of toxicological endpoints, including acute and chronic toxicity, carcinogenicity, genotoxicity, and hepatotoxicity, particularly when compounds are ingested repeatedly or in high concentrations. The presence of the chemical of concern affected negatively the overall scoring (− 1 point) of screened plant species. The table displaying the exact scoring for each plant species ( **Table 1S** , Supporting Information) enumerates the chemicals of concern. Notably, the compendium does not constitute a formal toxicological evaluation or regulatory classification of the safety status of the included botanical species. Instead, it functions as a preliminary screening tool to facilitate the identification of potential hazards, thereby supporting risk managers, regulatory authorities, food business operators, and researchers in their respective evaluative roles. The compendium presents botanical information in a structured tabular format, including data on botanical species, substances, or chemical groups of concern, the plant parts in which they were reported, preparations associated with possible concerns or adverse effects, and analytical methods, as well as measured concentrations. In the latest version, a separate plant genotoxicity table provides all relevant information from *in vitro* and *in vivo* genotoxicity studies associated with a specific plant or its plant part 58 . At the time of the evaluation (2024 and early 2025), information on the plant species in the online-available compendium comprised two key segments 59 . The upper segment, titled “Adverse Effect”, documents reported adverse effects associated with a given plant species that are not directly attributable to specific identified chemical constituents. If an entry is present in this section, the associated flag in the assessment is classified as “adverse effects not associated with chemicals of concern”. The lower segment, titled “Botanical Composition”, identified known constituents that are scientifically associated with potential risks for human health. In this case, the assessment is marked as “contains chemicals of concern”. In instances where both types of entries are present for a single plant species, the classification is recorded as “contains chemicals of concern and adverse effects not associated with them”. For the purposes of the present evaluation, the following scoring methodology was applied ( Table 2 ): a score of − 1 point was assigned to plants identified as containing chemicals of concern; a score of − 1 point was attributed to plants associated with adverse effects not linked to specific chemical constituents; a score of − 2 points was given to plants exhibiting both classifications simultaneously.

### BelFrIt list of plants allowed in food supplements

The BelFrIt List represents a tripartite regulatory initiative developed through collaboration between the national competent authorities of Belgium, France, and Italy, with the objective of harmonizing the national frameworks governing the use of botanicals in food supplements 60 . It serves as a non-binding reference document, compiling botanical species that are legally authorized for inclusion in food supplements within the territories of the participating countries. The initiative was established in response to the regulatory fragmentation that persists across the European Union due to Directive 2002/46/EC, which provides a general framework for food supplements but delegates the regulation of botanicals to individual member states 61 . This decentralized legislative structure has led to significant discrepancies regarding which plants are permitted in food supplements, the parts of the plants that are allowed, and their conditions of use. The BelFrIt List aims to mitigate such divergence by identifying species that are commonly authorized across the three countries, thereby promoting regulatory coherence and mutual recognition. Although the BelFrIt List does not constitute a harmonized EU standard, it functions as an important tool for regulatory alignment and risk management. It is widely used by regulators, manufacturers, and food business operators as a reference in the development and authorization of botanically based food supplements intended for international markets. Moreover, it contributes to the broader scientific discourse on the safety, traditional use, and regulatory treatment of botanicals within the European context and may serve as a supporting instrument in future harmonization efforts at the EU level in the fields of functional foods and dietary supplements. In the context of scientific or regulatory assessments–such as evaluating the suitability of a botanical candidate for inclusion in herbal medicinal products or food supplements–the presence of a species on the BelFrIt List can be interpreted as supportive evidence of its established traditional use and presumed safety profile, particularly when used under defined conditions. Within the current assessment framework, botanical species listed in the BelFrIt compendium were awarded 1 point, whereas those not included were assigned 0 points ( Table 2 ).

#### Status according to ComplʼAlim: French food supplements database

ComplʼAlim is a national database established and maintained by the French Agency for Food, Environmental, and Occupational Health and Safety (ANSES) 62 . It constitutes a key regulatory and scientific instrument that consolidates detailed information on food supplements notified for market placement in France. Developed within the framework of European Directive 2002/46/EC on food supplements, as transposed into French national legislation, the database aims to enhance transparency in the food supplement sector and support regulatory oversight through risk assessment and post-market surveillance activities. The database contains product-specific entries, including composition, intended use, manufacturer information, and regulatory notification status. As such, it serves as a critical tool for the French nutrivigilance system, facilitating the identification of potentially hazardous products and supporting evidence-based regulatory decisions. In the context of the present assessment, ComplʼAlim was integrated into the plant evaluation scoring system to determine the regulatory status of specific botanical species used in food supplements. The scoring was applied as follows ( Table 2 ): + 1 point was assigned if the plant and its specified conditions of use were explicitly authorized and listed in the database; − 1 point was applied if the plant or its intended use was listed as unauthorized or subject to restriction; 0 points were given if the plant was not referenced in the database.

#### Belgian plants order – List 1

Pursuant to the Royal Decree of 31 August 2021, commonly referred to as the Belgian Plants Order, a structured annex delineates three principal categories of botanical substances, among which List 1 enumerates plant species explicitly prohibited for use as, in, or as constituents of foodstuffs 63 . The enforcement of this regulatory instrument falls under the jurisdiction of the Federal Agency for the Safety of the Food Chain (FASFC), with expert input provided by the National Plant Preparations Advisory Committee. While embedded within the overarching framework of European Union food law, the Belgian Plants Order reflects the exercise of national regulatory autonomy in matters concerning botanicals. List 1 comprises plant taxa that are categorically banned from incorporation into food products, irrespective of their physical form (e.g., raw, dried, powdered, or extracted) or intended function (e.g., flavoring agent, ingredient, or food supplement component). The prohibition of these species is based primarily on toxicological risk considerations or the lack of adequate safety data in the context of food use. Inclusion of any plant listed in List 1 in foodstuffs or supplements constitutes a breach of Belgian food safety regulations and results in regulatory non-compliance. For the purposes of the present assessment, only List 1 was considered, while Lists 2 and 3, consisting of edible mushrooms and a list of plants that are allowed in food supplements (corresponding to the BelFrIt list), respectively, were excluded from analysis. Within the applied scoring methodology ( Table 2 ), plant species included in List 1 were assigned a score of − 1 point, whereas all other botanical species not appearing on this list were assigned a score of 0 points.

#### List of the products of plant (or animal) origin included in the normal human diet: Annex I of Regulation (EC) No. 396/2005

The “List of products of plant and animal origin included in the normal human diet”, as delineated in Annex I of Regulation (EC) No. 396/2005, constitutes a legally binding classification framework adopted by the European Union for the harmonized establishment and enforcement of maximum residue levels for pesticides in food and feed 64 . Annex I functions as the primary reference list of commodities for which pesticide residue thresholds are systematically defined under EU law. The products included are deemed representative of habitual dietary consumption within the Union and are thereby subjected to coordinated monitoring, risk assessment, and regulatory control. In the context of regulatory and scientific evaluations–particularly those concerning the use of botanical substances in food, feed, or veterinary applications–inclusion of a plant species in Annex I may serve as supporting evidence of established dietary exposure, widespread use, and regulatory acknowledgment. This, in turn, may influence risk stratification, guide the selection of appropriate monitoring strategies, and inform safety assessments related to pesticide residues. Within the framework of the present analysis, Annex I was employed as a proxy indicator for the consumer safety profile of plants proposed for use in herbal veterinary medicinal products. Specifically, the inclusion of a plant species in Annex I was interpreted as indicative of a favorable safety status due to its established use in the human diet. Accordingly, in the proposed scoring system ( Table 2 ), plant species listed in Annex I were assigned a score of + 1, while those absent from the Annex received a score of 0.

#### IUCN Red List status

The IUCN Red List status denotes the conservation classification of plant and animal species as determined by the International Union for Conservation of Nature (IUCN) within its globally recognized reference tool, the IUCN Red List of Threatened Species 65 . This resource represents one of the most authoritative and comprehensive databases on global species extinction risk, serving as a cornerstone for biodiversity monitoring, conservation planning, and policy development. Species are assigned to specific categories based on established quantitative criteria, including population size, rate of decline, geographical distribution, and the nature and intensity of threats. These categories include Extinct (EX), Extinct in the Wild (EW), Critically Endangered (CR), Endangered (EN), Vulnerable (VU), Near Threatened (NT), Least Concern (LC), Data Deficient (DD), and Not Evaluated (NE). In the context of evaluating botanical candidates for use in herbal veterinary medicinal products, the IUCN Red List status serves as a useful proxy for assessing ecological sustainability and the ethical acceptability of plant sourcing. For the purposes of the present assessment, a five-level scoring system was applied ( Table 2 ) to facilitate comparative analysis. Plant species classified as Least Concern were assigned a score of + 1, while those listed as Not Evaluated or Data Deficient were given 0 points. Species categorized as Near Threatened or Vulnerable received a score of − 1, those deemed Endangered or Critically Endangered were attributed − 2 points, and species listed as Extinct in the Wild or Extinct were assigned − 3 points. This scoring system enables the integration of biodiversity conservation principles into the selection process for HVMP candidates. Notably, it refers only to wildly growing plants and does not take into account the availability of plants cultivated by humans.

### Results of the screening procedure of medicinal plants as potential candidates for HVMPs

The screening of medicinal plants with previously documented veterinary use (ethnoveterinary data or employment in recently or previously registered HVMPs across Europe) resulted in a proposal for two lists of plant species. **Plant list A** ( Table 3 ) compiles 83 plant species that are sources of herbal substances/herbal preparations considered as the priority for a simplified registration procedure as HVMP in Europe, and which, overall, were scored positively (final score of more than 0). In several cases, this concerns specific parts and/or preparations only. In this context, it should be emphasized that the inclusion of a given plant species in the list of promising candidates does not obviate the need for comprehensive, evidence-based evaluation of the safety and efficacy of specific herbal preparations derived from that species. Such assessments remain essential, particularly when certain plant parts or their derivatives may contain compounds of toxicological concern (e.g., *β* -asarone in *Acorus calamus* ). It should, however, be noted that the presence of individual compounds of toxicological concern does not solely determine the overall toxicity profile of the whole plant. Indeed, accumulating evidence indicates that medicinal plants containing such constituents may still be used safely and effectively in animals, provided that appropriate consideration is given to factors such as plant part, preparation method, dosage, material quality, and exposure conditions 66 . **Plant list B** compiles plant species whose simplified registration is currently not considered as a priority or not advisable based on the available data; these plants are suggested to remain subjects of the currently existing registration procedures ( Table 4 ). The final score for those plants was 0 or less. However, their position in list A is not excluded if more data become available. The detailed scoring results for each plant species are presented in the Supporting Information ( **Table 1S** ).

**Table:** **Table 3** Plant List A: plant species whose herbal substances/preparations are considered a priority for a simplified registration procedure as HVMP in Europe.

Plant species	Assessment score
*Abies sibirica*	4
*Achillea millefolium*	11
*Acorus calamus*	5
*Aesculus hippocastanum*	9
*Aloe vera*	11
*Althaea officinalis*	14
*Anethum graveolens*	8
*Arctostaphylos uva-ursi*	8
*Arnica montana*	5
*Artemisia absinthium*	10
*Boswellia serrata*	7
*Calendula officinalis*	11
*Capsicum annuum*	11
*Carlina acaulis*	5
*Carum carvi*	14
*Centella asiatica*	13
*Cinnamomum cassia*	10
*Coriandrum sativum*	12
*Cupressus sempervirens*	7
*Cynara scolymus*	13
*Dysphania ambrosioides*	2
*Echinacea angustifolia*	13
*Echinacea pallida*	11
*Echinacea purpurea*	1
*Elymus repens*	8
*Equisetum arvense*	13
*Eucalyptus globulus*	15
*Fagus sylvatica*	2
*Foeniculum vulgare*	14
*Frangula alnus*	10
*Frangula purshiana*	9
*Gaultheria procumbens*	4
*Gentiana lutea*	16
*Glycyrrhiza glabra*	12
*Hamamelis virginiana*	13
*Harpagophytum procumbens*	9
*Hippophae rhamnoides*	5
*Hordeum vulgare*	5
*Humulus lupulus*	10
*Hypericum perforatum*	15
*Illicium verum*	7
*Juniperus communis*	15
*Larix decidua*	3
*Laurus nobilis*	10
*Lavandula angustifolia*	13
*Lespedeza capitata*	4
*Matricaria chamomilla*	15
*Medicago sativa*	8
*Melaleuca cajuputi*	5
*Melilotus officinalis*	8
*Melissa officinalis*	16
*Mentha x piperita*	15
*Monarda didyma*	4
*Myristica fragrans*	10
*Myroxylon balsamum*	7
*Ocimum basilicum*	6
*Origanum majorana*	12
*Orthosiphon aristatus*	8
*Phyllanthus emblica*	3
*Picea abies*	6
*Pilosella officinarum*	6
*Pimpinella anisum*	15
*Pinus sylvestris*	9
*Polygonum aviculare*	8
*Punica granatum*	9
*Quercus robur*	13
*Rheum palmatum*	11
*Rosa canina*	8
*Rosa rugosa*	2
*Ruscus aculeatus*	8
*Salvia officinalis*	14
*Salvia rosmarinus*	16
*Senna alexandrina*	11
*Sinapis alba*	5
*Symphytum officinale*	5
*Syzygium aromaticum*	13
*Tagetes erecta*	3
*Thymus vulgaris*	15
*Tilia cordata*	13
*Trigonella foenum-graecum*	11
*Urtica dioica*	14
*Valeriana officinalis*	11
*Vitellaria paradoxa*	1

As indicated in Table 3 , the highest-scoring plant species (≥ 14 points), including *Gentiana lutea, Salvia rosmarinus, Melissa officinalis* (16 points each), *Eucalyptus globulus, Hypericum perforatum, Juniperus communis, Matricaria chamomilla, Mentha × piperita, Pimpinella anisum* and *Thymus vulgaris* (15 points each), *Althaea officinalis, Carum carvi, Echinacea purpurea, Foeniculum vulgare* , and *Urtica dioica* (14 points each), can be regarded as priority candidates for assessment for simplified registration of herbal veterinary medicinal products (HVMPs). Their extensive regulatory recognition, pharmacopoeial inclusion, and well-documented safety and efficacy profiles underscore both their broad therapeutic potential and their appropriateness for evidence-based veterinary practice. Moreover, their biological and pharmacological activities were confirmed in regulatory dossiers and numerous ethnoveterinary studies 27 , 42 , 43 (details are presented in **Table 1S** , Supporting Information). Careful analysis of list A reveals that 36 species (i.e., *Arctostaphylos uva-ursi, Artemisia absinthium, Boswellia serrata, Carlina acaulis, Cynara scolymus, Dysphania ambrosioides, Elymus repens, Equisetum arvense, Fagus sylvatica, Frangula purshiana, Gaultheria procumbens, Glycyrrhiza glabra, Hippophae rhamnoides, Hordeum vulgare, Humulus lupulus, Illicium verum, Larix decidua, Melaleuca cajuputi, Melilotus officinalis, Monarda didyma, Myroxylon balsamum, Ocimum basilicum, Orthosiphon aristatus, Phyllanthus emblica, Pilosella officinarum, Pinus sylvestris, Polygonum aviculare, Punica granatum, Rosa canina, Rosa rugosa, Senna alexandrina, Sinapis alba, Tagetes erecta, Trigonella foenum-graecum, Valeriana officinalis* , and *Vitellaria paradoxa* ) were found to be promising candidates, although they are not included in Table 1 of Commission Regulation (EU) No. 37/2010.

**Table:** **Table 4** Plant List B: plant species for whose products simplified registration is not currently deemed a priority or advisable based on the available data.

Plant species	Assessment score
*Areca catechu*	0
*Asarum europaeum*	− 4
*Baptisia tinctoria*	− 4
*Canarium luzonicum*	− 1
*Cedrus atlantica*	− 2
*Chelidonium majus*	− 1
*Croton geayi*	− 1
*Croton tiglium*	− 3
*Derris elliptica*	− 3
*Digitalis purpurea*	0
*Dryopteris filix-mas*	− 3
*Mallotus philippensis*	− 5
*Oenanthe aquatica*	0
*Ouret lanata*	0
*Phytolacca americana*	− 2
*Pulsatilla pratensis*	− 3
*Sassafras albidum*	− 1
*Securidaca longepedunculata*	0
*Thuja occidentalis*	0
*Veratrum album*	− 3
*Veratrum lobelianum*	− 2

Of particular relevance, in some instances, the outcomes of the initial assessment must be interpreted in light of animal species-specific toxicology and the risk–benefit profiles of each animal species. Consequently, further refinement remains essential to validate the suitability of individual plant species for veterinary medicinal applications in the various treated animal species. Such a refinement might include expert experience and judgement, as well as historical or ethnoveterinary data, or, wherever available, recent animal species-specific toxicological data. It may happen that medicinal plants are initially identified as promising candidates; however, due to specific unresolved concerns or data limitations, they ultimately need further assessment. The complexity of the assessment is exemplified by the case of *Thymus vulgaris* (scored very high, with a final result of 15), which is recognized as part of the normal human diet and is also known for its potential toxicity in carnivore companion animals, such as felines 44 , 67 , 68 . Nonetheless, a veterinary medicinal product containing extracts from *Thymus vulgaris* has been used for decades and was recently registered for non-carnivorous species. This highlights the need to balance therapeutic potential with species-specific safety concerns, particularly in carnivore species.

### Development of a multi-criteria framework for the identification of medicinal plants for simplified HVMP registration

To facilitate the systematic identification of promising botanical candidates suitable as sources of herbal active substances for simplified registration as HVMPs, a scientifically robust and multidimensional evaluation framework was developed. This framework incorporates a comprehensive range of authoritative sources, including regulatory compendia, pharmacopoeial references, and scientific monographs. The selection criteria for these sources were grounded in their demonstrated relevance to key domains, including ^safety, efficacy, traditional use, regulatory precedence, and ecological sustainability, of plant-derived substances intended for veterinary use.

The adoption of such a structured, evidence-based approach provides a rational, responsible methodology for screening and prioritizing plant species for HVMP development. This aligns particularly with the European Unionʼs objectives of fostering innovation in veterinary medicinal products, while adhering to the principles of the One Health paradigm and sustainability commitments. By leveraging existing bibliographic data and pre-validated sources, the framework supports regulatory simplification without compromising scientific or public health standards. The core of the proposed framework lies in its ability to consolidate and interpret pre-existing data on safety and efficacy. Sources such as EMA/HMPC, WHO, and ESCOP 48 – 53 monographs provide scientifically vetted documentation of traditional or well-established medicinal uses in humans. These sources are particularly valuable under the traditional use registration pathway, which permits reliance on bibliographic evidence in lieu of extensive preclinical and clinical studies. This not only expedites the dossier preparation process but also reduces the cost and time associated with product development. Regulatory harmonization and mutual recognition across jurisdictions are further supported through reference to sources such as Regulation (EC) No. 37/2010 45 , the BelFrIt list 60 , and the ComplʼAlim database 62 . The inclusion of a botanical substance in such sources implies that it has undergone prior regulatory evaluation and acceptance for either medicinal or food use purposes. This reinforces regulatory predictability and streamlines the assessment process by reducing the need for *de novo* justification of known substances. In terms of pharmaceutical quality and compliance, the framework references the European Pharmacopoeia, which provides standardized monographs detailing identification, purity testing, and assay methodologies for botanical materials. This facilitates adherence to good manufacturing practice (GMP) and minimizes the evidentiary burden on manufacturers regarding quality specifications. Additionally, European legislation mandates that efficacy, toxicity, and residual studies be conducted in accordance with good laboratory practice (GLP) standards for each relevant animal species. Risk mitigation is addressed through pre-screening using toxicological databases such as the EFSA Compendium of Botanicals 59 and national negative lists, including the Belgian list of prohibited plants 63 . These tools facilitate the early elimination of botanicals with known toxicological profiles, thereby focusing development efforts on lower-risk candidates and reducing the likelihood of rejection during regulatory evaluation. The inclusion of national and international registers of traditional use, such as the French list of traditionally used medicinal plants and the WHO monographs, enhances the evidence base for simplified registration routes, which inherently require a lower evidentiary threshold than full marketing authorizations. Concurrently, the assessment incorporated the critical perspective of veterinary pharmacologists to ensure that species-specific pharmacodynamics, pharmacokinetics, and interspecies variability were adequately addressed. Sustainability is integrated via the IUCN Red List of Threatened Species 65 , which provides a standardized global assessment of the conservation status of wild species. Incorporating this dimension ensures that botanical candidates never contribute to the depletion of natural resources. It also aligns the framework with broader environmental and ethical objectives, reinforcing its suitability under emerging green and One Health–oriented veterinary policies. Finally, it is important to note that efficacy was not evaluated within the scope of the assessment, as the selection of medicinal plants screened was based on a predefined list comprising species that are currently, or have previously been, components of registered HVMPs.

### Limitations and considerations

First, it should be emphasized that the primary objective of the proposed assessment protocol was to verify the suitability of the screened medicinal plants for consumer and end-user safety. In contrast, comparatively limited emphasis was placed on animal safety and therapeutic efficacy, which nevertheless constitute equally critical pillars of the authorization process for HVMPs. Despite its strengths, several limitations inherent to the proposed methodology warrant critical attention. A primary concern is the predominance of human-healthcare-derived data within core reference sources such as the HMPC/EMA, WHO, and ESCOP monographs 48 – 53 . The extrapolation of these data to veterinary contexts requires careful scientific justification, particularly given interspecies differences in pharmacodynamics, metabolism, and therapeutic indications. This might be explicitly true for animals like carnivores, which, given their evolutionary background, have a lower need to cope with bioactive plant components than humans and pigs, as omnivores, or horses and ruminants, as herbivores. Moreover, the comprehensiveness and harmonization of the referenced databases vary significantly. Also included national inventories, such as, for example, the BelFrIt or ComplʼAlim lists, although informative, lack binding legal status at the EU level. This may result in regulatory uncertainty or inconsistency when developing products intended for distribution in multiple member states. The BelFrIt list, the ComplʼAlim database, and the Belgian Plants Order 60 , 62 , 63 represent three distinct but related instruments governing the use of botanicals in food supplements. The BelFrIt list, jointly developed by Belgium, France, and Italy, constitutes a harmonized positive list of plant species (including plant parts and specific preparations) authorized for use in food supplements. It defines permitted plant parts, conditions of use, maximum levels, and mandatory labeling requirements, thereby fostering regulatory convergence among the three countries. In contrast, ComplʼAlim is a French national digital platform that goes beyond listing substances: it manages the notification and compliance procedures for food supplements, integrates ingredient validation, enables dossier submission, and provides regulatory checks, serving as a practical operational tool to ensure conformity of products marketed in France. The Belgian Plants Order (Royal Decree of 1997 and subsequent amendments), however, is more restrictive in nature: it establishes both a positive list of plants authorized for use and a negative list of prohibited or restricted plants, reflecting Belgiumʼs precautionary approach to consumer protection. In practice, the BelFrIt list offers a harmonized scientific reference. ComplʼAlim functions as a regulatory and administrative control mechanism in France, and the Belgian Plants Order provides a legally binding framework that delineates which plants are permitted or excluded from food supplements on the Belgian market. Together, these instruments illustrate the diversity of national and regional approaches to regulating botanicals in food supplements within the EU.

Additionally, plants with regionally important but poorly documented traditional uses may be excluded, potentially omitting valuable candidates from consideration. The temporal limitations of the data sources must also be acknowledged. Many compendia are updated infrequently and may not reflect the most recent advancements in toxicological or pharmacological knowledge. Consequently, relying solely on these sources could perpetuate outdated risk assessments or overlook newly validated substances. Furthermore, less commercially prominent or underutilized species are often underrepresented in mainstream databases. For such species, limited supporting data may impede their qualification under simplified registration procedures, despite potential efficacy or sustainability advantages. Conversely, adopting updated versions of the databases underpinning the present assessment model may introduce variability in the resulting scores. For instance, the EFSA Compendium of Botanicals underwent its most recent revision on 12 May 2025 58 , subsequent to the completion of our assessment. Looking forward to the perspectives of HVMPs, it is worth noting that, in addition to pre-clinical, clinical, and regulatory safety data, information from post-marketing surveillance systems and documented off-label uses should be considered, as these may provide valuable insights into rare adverse events, long-term safety, and species-specific risks.

The proposed evaluation framework represents a structured, science-driven instrument designed to inform and optimize the selection of botanical candidates for assessing simplified HVMP registration. It is designed to serve as a quick, pragmatic screening process for medicinal plants as sources of herbal active ingredients and has been tested on more than 100 plant species. The next assessment phase should include defining the specific herbal substances/preparations derived from selected plant candidates eligible for simplified registration. While it serves as a valuable screening tool, its application should be seen as part of a broader, iterative decision-making process. It would be highly advantageous if the conclusions derived from the present analysis, along with the methodological insights gained, were taken into consideration in the context of developing the implementation framework or policy response to Article 157 of Regulation (EU) 2019/6 35 : “The Commission shall report to the European Parliament and to the Council by January 29, 2027, on traditional herbal products used to treat animals in the Union. If appropriate, the Commission shall submit a legislative proposal to introduce a simplified system for registering traditional herbal products used to treat animals. The Member States shall provide information to the Commission on such traditional herbal products within their territories.”

## Footnotes

1 Pure substances of typically synthetic origin

2 The term “herbal substance” is defined as “herbal drug” in the European Pharmacopoeia.

## Supplementary Material

The results of the inclusion screening and the evaluation, with detailed scoring for each plant species, are presented in the Supporting Information ( **Table 1S** ).

Supplementary Material Zusatzmaterial
